# Polygenic risk scores for disease risk prediction in Africa: current challenges and future directions

**DOI:** 10.1186/s13073-023-01245-9

**Published:** 2023-10-30

**Authors:** Segun Fatumo, Dassen Sathan, Chaimae Samtal, Itunuoluwa Isewon, Tsaone Tamuhla, Chisom Soremekun, James Jafali, Sumir Panji, Nicki Tiffin, Yasmina Jaufeerally Fakim

**Affiliations:** 1 The African Computational Genomics (TACG) Research Group, MRC/UVRI and LSHTM, Entebbe, Uganda; 2 H3Africa Bioinformatics Network (H3ABioNet) Node, Centre for Genomics Research and Innovation, NABDA/FMST, Abuja, Nigeria; 3 Department of Non-Communicable Disease Epidemiology (NCDE), London School of Hygiene and Tropical Medicine, Keppel St, London, WC1E 7HT UK; 4 H3Africa Bioinformatics Network (H3ABioNet) Node, University of Mauritius, Reduit, Mauritius; 5 Laboratory of Biotechnology, Environment, Agri-Food and Health, Faculty of Sciences Dhar El Mahraz–Sidi Mohammed Ben Abdellah University, 30000 Fez, Morocco; 6 Department of Computer and Information Sciences, Covenant University, P. M. B. 1023, Ota, Ogun State Nigeria; 7 Covenant University Bioinformatics Research (CUBRe), Covenant University, Km 10 Idiroko Road, P.M.B. 1023, Ota, Ogun State Nigeria; 8 Covenant Applied Informatics and Communication African Centre of Excellence (CApIC-ACE), Covenant University, P.M.B. 1023, Ota, Ogun State Nigeria; 9 Division of Computational Biology, Integrative Biomedical Sciences Department, Faculty of Health Sciences, University of Cape Town, Cape Town, South Africa; 10 South African Medical Research Council Bioinformatics Unit, South African National Bioinformatics Institute, University of the Western Cape, Bellville, South Africa; 11 Department of Immunology and Molecular Biology, College of Health Science, Makerere University, Kampala, Uganda; 12 Malawi-Liverpool-Wellcome Trust Clinical Research Programme, Blantyre, Malawi; 13 Clinical Infection, Microbiology & Immunology, The University of Liverpool, Liverpool, UK; 14 Computational Biology Group, Department of Integrative Biomedical Sciences, Institute of Infectious Disease and Molecular Medicine, Faculty of Health Sciences, University of Cape Town, Cape Town, 7925 South Africa

**Keywords:** Polygenic risk scores, Africa, Genetic diversity, Genomic architecture, Complex diseases

## Abstract

Early identification of genetic risk factors for complex diseases can enable timely interventions and prevent serious outcomes, including mortality. While the genetics underlying many Mendelian diseases have been elucidated, it is harder to predict risk for complex diseases arising from the combined effects of many genetic variants with smaller individual effects on disease aetiology. Polygenic risk scores (PRS), which combine multiple contributing variants to predict disease risk, have the potential to influence the implementation for precision medicine. However, the majority of existing PRS were developed from European data with limited transferability to African populations. Notably, African populations have diverse genetic backgrounds, and a genomic architecture with smaller haplotype blocks compared to European genomes. Subsequently, growing evidence shows that using large-scale African ancestry cohorts as discovery for PRS development may generate more generalizable findings. Here, we (1) discuss the factors contributing to the poor transferability of PRS in African populations, (2) showcase the novel Africa genomic datasets for PRS development, (3) explore the potential clinical utility of PRS in African populations, and (4) provide insight into the future of PRS in Africa.

**Supplementary Information:**

The online version contains supplementary material available at 10.1186/s13073-023-01245-9.

## Background

Disease prevalence varies largely across the world, and some diseases are often specific to certain geographic locations. Lifestyle, diet, and environmental determinants, as well as genetic factors, explain pathological conditions in diverse settings and are likely to impact on the severity in different individuals and populations (https://www.who.int/data/gho,18/10/2022). Clinical risk can be evaluated from the analysis of blood biomarkers, symptoms, and prevailing family history. However, recent work has suggested that risk prediction for common chronic diseases can be improved using genetic data [1].

**Fig. 1:**
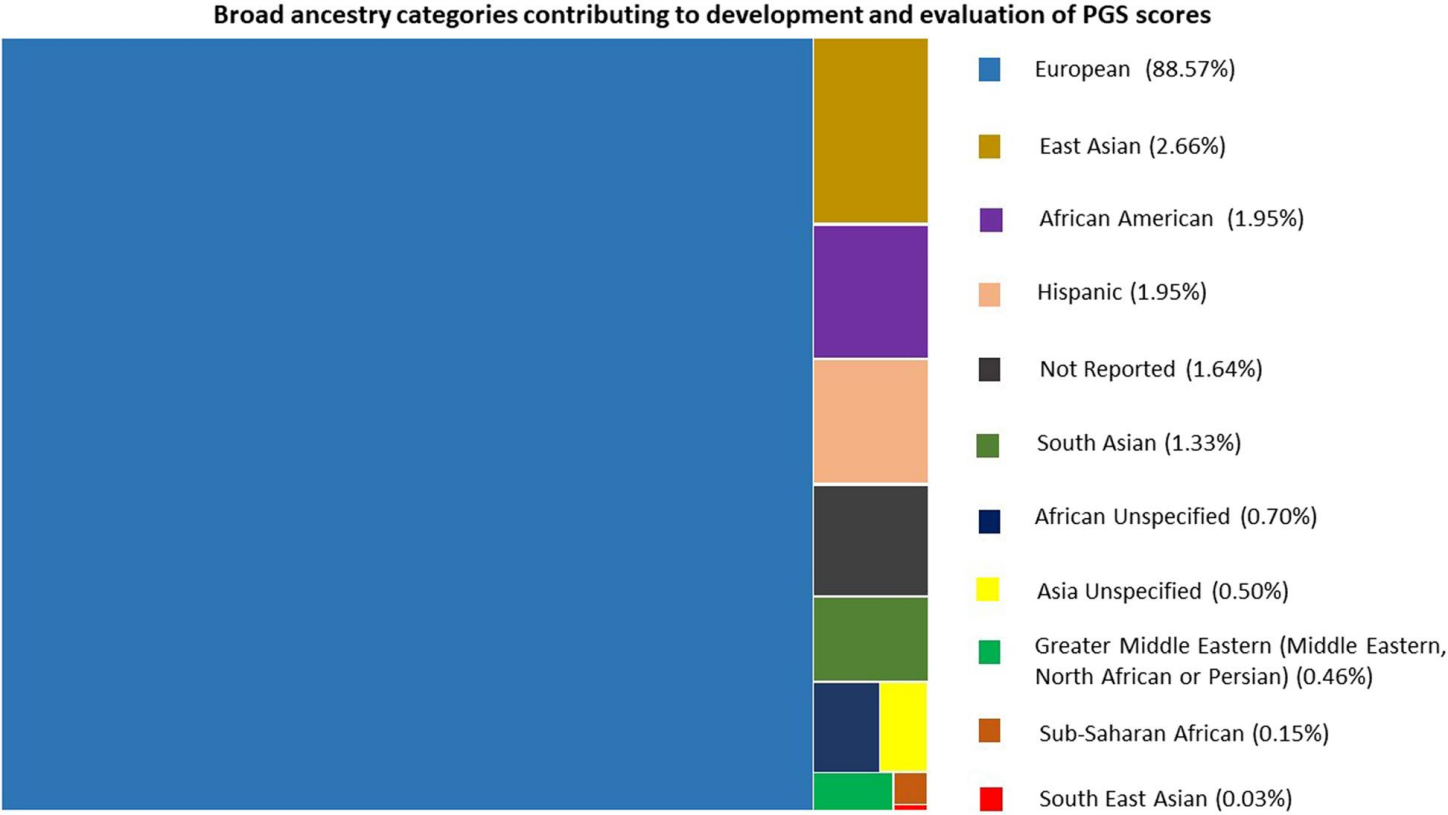
Proportion of broad ancestry populations that contributed to the development and evaluation of 2555 PGS scores within the PGS Catalogue (version 2023–06-23). African unspecified: African that could not be classified as African American, Afro-Caribbean, or Sub-Saharan African. Asian unspecified: Asians that could not be classified as East Asian, Central Asian, South Asian, or South-East Asian. Multiple studies and subsets of data can contribute towards the development and evaluation of a PGS scores within the PGS Catalogue

**Fig. 2:**
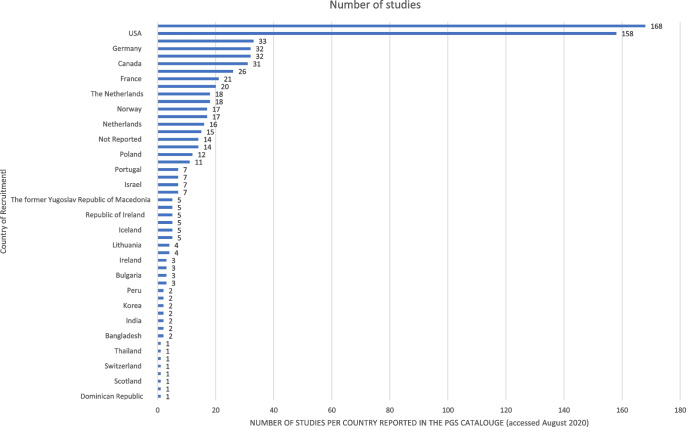
Count of reported countries of recruitment for samples included in the PGS Catalogue (accessed August 2020)

Genome-wide association studies (GWAS) have significantly contributed to identifying a huge number of loci associated to a variety of complex diseases and traits. However, most genetic association discoveries have been made in European ancestry individuals [2–5]. The strength of the genetic association with phenotypes is enhanced when phenotypic data is available from large-scale studies linked with relevant phenotypic data and electronic health records. Recently, polygenic risk scores (PRSs), which weigh the genetic effect of numerous common variations associated to disease or traits, have gained popularity to quantify an individual’s genetic risk for a disease or trait. The pace of research in this area has recently improved, and PRS scores are now available for a variety of traits and diseases, mostly in the European population (Figs. 1 and 2). As a result, PRS is quickly becoming a common tool for estimating genetic liability in predicting disease risks, which is essential for early disease identification, prevention, and intervention.

The poor transferability of PRS derived from European ancestry dataset to diverse African populations is a cause of concern. This is likely to be due to unique differences in genetic architecture and environmental exposures of the different populations [4]. The lack of accurate PRS in African ancestry individuals may cause barrier to achieve precise risk stratification which is critical for precision medicine. Given that the human genetic diversity is greater in Africa, and when large-scale African ancestry cohorts are available for the development of PRS, this may generate more generalizable findings [6]. This is high importance, not only for Africa but for the entire global medical and research community. For example, the identification of *PCSK9* missense mutations and their impact on plasma low-density lipoprotein cholesterol levels across diverse ancestries. This breakthrough discovery exemplifies how African ancestry individuals have contributed to advancing medical knowledge, thereby benefiting the entire human race. The rich genetic variation in African populations provides so many opportunities that extends well beyond the scope of PRS. In this review article, the aim is to (1) review factors contributing to poor transferability of PRS in African populations, (2) showcase the novel genomic datasets that could enhance PRS transferability in continental Africa, and (3) explore the potential clinical utility of PRS in African populations.

## Factors contributing to poor transferability of PRS in African populations

There are many factors contributing to poor transferability of PRS in African populations. This includes genetic factors such as minor allele frequencies, difference in linkage disequilibrium patterns, and their interactions with environmental considerations like diet, exercise, age, gender, and variability in phenotype measurement.

PRS are calculated by aggregating the effect of many common variants that are associated with the diseases of interest. Given that Africa is the continent where all humans originated, it has the highest genetic diversity in the whole world. However, the current lack of diversity in genomic studies have implications on the predictive power of the methods that are trained and developed on euro-centric datasets. PRS constructed with such method may differ primarily on how the weight of the effect size is generated and how the number of single nucleotide polymorphism (SNPs) to be included in the PRS calculation is determined. For example, the interrogation of high-risk variants may involve inclusion of a causal variant from a population, whereas PRS estimates may incorporate variants that are not perfectly correlated with the causal genetic factor [7]. The implication of this is that the method may incorporate a variant with uncertain effect size in the PRS which invariably may reduce the generalizability of PRS risk estimates in the target population.

A linkage disequilibrium (LD) reference panel and data on allele frequencies are prerequisites for application of PRS methods in a heterogeneous background. These factors are important for PRS development. For example, allele frequency differences may cause predicted risks of a disease to vary across populations. Given that LD blocks are shorter in African populations, the SNPs which are in high LD can be removed as they inflate the score. Several studies have shown lower levels of LD in African populations compared to other populations [8] which may imply that the power to detect untyped causal loci is reduced. This LD and distance between the causal variants and the GWAS tagging SNPs can explain lower accuracy and limited transferability. The relative accuracy of polygenic scores is enhanced when LD and minor allele frequencies are integrated into the model [9] assuming that causal variants are shared between populations. Invariably, LD pattern differences between discovery and target populations may impact the effect size calculation and determination of causal variant. Therefore, the transferability of risk score across populations is a major challenge when they do not share the same genetic architecture for each disease [10].

The phenomenon of pleiotropy is an indication of the complexity of how mutations in one locus may influence several pathways or functions. One gene may be responsible for different unrelated traits. SNP markers that are selected for PRS calculations may well impact on another phenotype(s) than the one for which the risk is being calculated. Graff et al. found patterns of pleiotropy when investigating PRS in Europeans for 16 different types of cancers [11]. Positive associations were reported between several forms of cancer. Some variants may be associated with a disease while being protective for another pathology [11]. Pleiotropic effects of certain variants may well confound risk estimations when used for PRS calculations [11].

Many PRS methods have attempted to solve the problem of different LD pattern using LD clumping or/and penalised regression. Ge and colleagues [12] and Baker and colleagues [13] have extensively reviewed such different PRS methods, and, as such, this paper is not intended to duplicate this effort. However, we provide a summary of how some popular PRS tools account for LD (Additional file 1: Table S1). The main problem with the clumping and thresholding approach is that it does not take environmental factors such diet and exercise into consideration which might confound the predictive accuracy of these measures.

In addition to genetic contributions to lower PRS accuracy in African population, environmental exposures can also play a major role in contributing to poor transferability of PRS across populations [5]. As most GWAS may have already been subject to ascertainment bias [7], such study recruitment mainly from rural, urban, healthier, poorer, or educated participants only may introduce collider bias [14]. In a recently published paper describing PRS in African populations, Kamiza and colleagues^4^ show that environmental factors such as diet, exercise, age, gender, and living in rural or urban community can influence PRS portability [15]. The results from this paper suggest that poor transferability of PRS between South African Zulu and Ugandan populations is due to differences in environmental and genetic factors between the two African populations [15]. They also showed that lipid predictability was lower in East Africa Uganda population than in South Africa Zulu population which was attributed to non-fasting of participants before blood collection for lipid analysis. Similarly, a type 2 diabetes PRS paper [16] shows a varied predictability between Kenya and Ghana and Nigeria, where predictability was much higher. Although these two studies show that PRS derived from data of African American individuals enhance polygenic prediction in sub-Sahara Africa compared to European and multi-ancestry scores, it is important to note that the studies further show that PRS prediction varied greatly within SSA, implying that African American-derived PRS may not be generalizable across populations in Africa. This reason may be that only certain geographies and genetic variation are represented in African American [17].

In the study by Reisberg and colleagues [18], SNPs from the European cohorts were used for calculating the risk for type 2 diabetes, and the 1000 Genomes dataset of African populations had the highest scores compared to Europeans. The report from Hugues and colleagues^15^ verifies that when population-specific SNPs are included in the calculations, the risk calculation is improved. Considerable efforts to understand GxE interaction effects is key for transferability of PRS as the effects of genetic variants on phenotype can be different between populations as demonstrated by Chikowore and colleagues [16].

In addition, research strategies and medical procedures are not always consistent across all countries in Africa. This is critical for diseases such as psychiatric disorders, where phenotype reporting requires intricate and complicated procedures. As an alternative, minimal phenotyping, which has recourse to hospital records, self-reporting symptoms, or prescription of medications, is used for identification of cases. This approach consists of sampling based on heterogeneous self-reported symptoms and not on the recommended criteria for diagnosis. GWAS based on minimal phenotyping produce a large number of associated loci which are however of lower heritability and have non-specific effects. Cai et al. show that when minimal phenotyping is used, for major depressive disorder (MDD), the genetic architecture is different from when the strictly defined MDD is used [19].

Collectively, genetic factors such as differences in effect sizes, allele frequencies, LD patterns, phenomenon of pleiotropy, and phenotyping in addition to environmental exposures are limiting the generalizability of genetic predictions of diseases and traits to African populations. Pereira, et al. discussed in detail these factors that influence PRSs and limit transferability including highlighting the complex scenarios of the importance of using genomic data from multiple populations to develop appropriate population-specific applications [20].

### Growing collection of continental African genomic datasets for more accurate PRS

To do genomic research, biobanks are essential. As a result, national biobanks have been established by several governments globally to support scientific research and advance precision medicine. One notable example is UKBioBank [21], a well-known biobank that gathers health and genetic information from 500,000 people in the UK. (2) The All of Us Research Programme (USA) seeks to recruit one million or more individuals from a variety of backgrounds to provide a resource for precision medicine [22]. (3) The Estonian Biobank—a nationwide biobank effort with the aim of enhancing genetic research and healthcare in the nation—collects genomic and health-related data from over 200,000 members [23]. Some other genomic medicine initiatives include those from Canada, Qatar, Turkey, Japan, Finland, Denmark, Australia, Saudi Arabia, Switzerland, China, and Brazil [24], but such national biobank is lacking in Africa.

Growing evidence shows that using large-scale African ancestry cohorts as discovery for PRS development may generate more generalizable findings. Data from GWAS are fundamental as they are used for developing PRS. To date, GWAS has increasingly identified a large number of genetic variants which are associated with a range of complex traits [5, 7, 24, 25]. However, the majority of GWAS has been conducted with data from individual of primarily European and Asian descents [5, 25]. PRS can help to estimate individual’s genetic risk to a disease or condition by aggregating the effect of many common variants associated with the condition, but studies have shown that well-powered large-scale-based data are required to derive PRS which are currently lacking in continental Africa. This calls for the need to initiate a step-change in the scale of such studies in African populations to enhance PRS prediction or aggregate emerging genomic datasets comparable with European and Asian genomic initiatives. African genetic data have revealed highly relevant African-enriched variants in genes such as *APOL1*, PCSK9, and *G6PD* for kidney diseases, lipid traits, and diabetes respectively [26]. In Table 1, we show a growing collection of rich continental African genomic datasets linked to mostly non-communicable disease phenotypes becoming available for generating PRS for African populations.

**Table 1:** Description of a range of genomic datasets in Africa for potential polygenic risk scores for disease risk in Continental Africa

Full name (Study)	Country	Ancestry/ethics	Number of samples	Disease/traits	Lifestyle/exposure variables	Genotyping platform	Reference	Controlled access on EGA/DbGAP/ accession number
Uganda Genome Resource (UGR)	Uganda	Ugandans	7 000	Blood Pressure, lipid, Anthropometries, Full blood count, Liver Function, HbA1c, Kidney function, others	smoking, diet, physical activity, others	Illumina HumanOmni 2.5 M BeadChip array	[27, 28]	EGA: EGAD00010000965; EGAD00001001639
Durban Diabetes Study (DDS)	South Africa	South African Zulu	1,200	Diabetes Mellitus, BP, lipid, Anthropometries, Full Blood Counts, Liver Function	smoking, diet, physical activity, others	Customised Illumina Multi-Ethnic Genotyping Array (MEGA)	[29]	Not known
Durban Diabetes Case Control study (DCC)	South Africa	South African Zulu	1 600	Diabetes Mellitus, BP, lipid, Anthropometries, Full Blood Counts, Liver Function	smoking, diet, physical activity, others	Customised Illumina Multi-Ethnic Genotyping Array (MEGA)	[29]	Not known
Africa America Diabetes Mellitus (AADM)	Nigeria, Ghana & Kenya	Sub-Saharan African	5 000	Diabetes Mellitus, BP, lipid, Anthropometries, Full blood count, Liver Function, Kidney function, others	smoking, diet, physical activity, others	Affymetrix Axiom PANAFR SNP array	[30]	dbGAP: phs001844
Research on Obesity and Diabetes among African Migrants (RODAM and HELIUS)	Ghana, Suriname, Morocco	Africans	13 655	Diabetes Mellitus, BP, lipid, Anthropometries, Kidney Function, others	smoking, diet, physical activity, others	Not Known	[31]	EGA: EGAD00001004106
Muhimbili Sickle Cohort (Haemoglobin GWAS study)	Tanzania	Tanzania	1213	Sickle cell disease, Anthropometries, Others		Illumina Omni2.5 array	[32]	Not known
Genomic and Environmental Risk Factors for Cardiometabolic Disease in Africans (AWI-GEN)	Burkina Faso, Ghana, Kenya and South Africa	Sub-Saharan African	12 000	Blood Pressure, lipid, Anthropometries, Full blood count, Liver Function, Kidney function, others	smoking, diet, physical activity, others	H3Africa Illumina Array	[33, 34]	EGA EGAD00001004448
H3Africa Diabetes study (H3Africa Diabetics)	Various across Sub-Sahara Africa	Sub-Saharan African	12 621	Diabetes Mellitus, BP, lipid, Anthropometries, others	smoking, diet, physical activity, others	Planned H3Africa Illumina Array	[35]	Not Known
Genetics of rheumatic heart disease (RHDGen) Network	Various across Sub-Sahara Africa	Sub-Saharan African	3 555	rheumatic heart disease, BP, lipid, Anthropometries, others		Planned H3Africa Illumina Array	[36]	Not known
African Collaborative Center for Microbiome and Genomics Research (ACCME)	Nigeria	Nigerian	11 700	cervical cancer, Blood Pressure, lipid, Anthropometries, others	smoking, diet, physical activity, others	H3Africa Illumina Array	[37]	dbGAP phs001945
African Female Breast Cancer Epidemiology (AFBRECANE)	Nigeria	Nigerian	1 000	Breast Cancer, Blood Pressure, lipid, Anthropometries, others	smoking, diet, physical activity, others	Not Known	[38]	Not known
KEMRI VaccGene study	Kenya	Kenya	1 400	Blood Pressure, Anthropometries, others	smoking, diet, physical activity, others	Not Known	[39, 40]	Not known
Malawi Genome Resource	Malawian	Malawian	6 626	Diabetes Mellitus, BP, lipid, Anthropometries, Kidney Function, others	smoking, diet, physical activity, others	H3Africa Illumina Array version 2	[41]	Not known
The Stroke Investigative Research and Educational Network (SIREN)	Nigeria	Nigerian	8 000	Stroke, Blood Pressure, Anthropometries and others	smoking, diet, physical activity, others	H3Africa Illumina Array	[42]	Not known
Trauma and PTSD study	Rwanda	Rwandans	400	Trauma, Post-traumatic stress disorder (PTSD), Blood Pressure, Anthropometries and others	smoking, diet, physical activity, others	Planned H3Africa Illumina Array	[43, 44]	Not known
Keneba Biobank	Gambia	Gambians	10 000	Blood Pressure, serum creatinine, Anthropometries and others	smoking, diet, physical activity, others	Planned H3Africa Illumina Array	[45]	Not known
The Sickle Cell Disease Genomics of Africa (SickleGenAfrica)	Cameroon, Ghana, Nigeria and Tanzania	Africans	7 000	Sickle cell disease, Blood Pressure, Anthropometries and others	smoking, diet, physical activity, others	H3Africa Illumina Array	[46]	Not known
The H3Africa Kidney Disease study	Ghana, Nigeria, Ethiopia, and Kenya	Africans	11 964	Kidney function, Blood Pressure, Anthropometries and others	smoking, diet, physical activity, others	Planned H3Africa Illumina Array	[47]	EGA EGAD00010002365; EGAD00001009333
Collaborative African Genomics Network (CAfGEN)	Botswana, Uganda, and Swaziland	Africans	1 000	HIV, Blood Pressure, Anthropometries and others	smoking, diet, physical activity, others	Illumina HiSeq 2500	[48]	EGAD00001006224
The Tanzania Stroke Incidence Project (TSIP)	Tanzania	Tanzanian	636	Stroke, Anthropometries, Others		Not Known	[49]	Not known
Non-Communicable Diseases – Genetic Heritage Study (NCD-GHS)	Nigeria	Nigerian	100 000	Blood Pressure, lipid, Anthropometries, Full blood count, Liver Function, HbA1c, Kidney function, Cancer, Diabetes Mellitus others	smoking, diet, physical activity, others	Illumina Global Array	[50]	Not known
H3AFRICA TRYPANOGEN PROJECT	Uganda Guinea Ivory Coast Burkina Faso Cameroon DRC Malawi Zambia	Africans	233	trypanosomiasis	lifestyle	Illumina HiSeq 2500	[51]	EGAS00001002602
H3Africa Genomic Characterization and Surveillance of Microbial Threats in West Africa	Nigeria, Sierra Leone	West Africa	2667 1345	Lassa Fever	lifestyle	Illumina Omni 2.5 M, Illumina Omni 5 M	[52]	EGAD00010002509
NeuroGAP-Psychosis	Kenya, Ethiopia, Uganda, South Africa	South and East Africans	17,000	Schizophrenia, Bipolar disorder	lifestyle	Global Sequencing Array	[53]	dbGaP accession phs002528.v1.p1
NeuroDev project	Kenya, South Africa	South and East Africans	5,000	Autism spectrum disorder (ASD), Intellectual disability (ID), Attention deficit hyperactivity disorder (ADHD)	lifestyle	Global Sequencing Array	[54]	dbGap accession number phs001874
The African Biobank	Nigeria, Kenya, Tanzania, Zimbabwe and South Africa	Africans	1,488	Pharmacogenetics	lifestyle	PCR–RFLP	[55]	Not known
MalariaGEN	Burkina Faso, Cameroon, Ghana, Kenya, Malawi, Mali, Nigeria, Papua New Guinea, Tanzania, The Gambia, Vietnam	Africa, Asia and Oceania	17,000	Malaria	lifestyle	Sequenom MassArray platform	[56–58]	EGAS00000000026
***Total***	**262,303**							

One key factor to determine the accuracy and predictive power of PRS is the power of the discovery GWAS data to avoid reaching misleading conclusions [9]. To improve cross-population polygenic risk prediction, specifically, Weissbrod and colleagues [59] recommended that base GWAS should have at least 100 K individuals to observe relate prediction and accuracy of PRS. Unfortunately, the most current GWAS data from continental Africa are under-powered with sample sizes ranging from 150 to 12,000 individuals representing only 1.1% of genomic studies from all African ancestry individuals [5].

In order to improve the representation of African genomic data in the global context for discovery and genetic risk prediction in the last decade, some initiatives have been initiated in Africa including the Africa America Diabetes Mellitus (AADM) [30], the Uganda genome project [27, 28], the Human Heredity and Health in Africa consortium [60, 61], the Nigerian 100 K genome project [50], and many others with smaller sample sizes and limited potential to get published. Aggregating all the datasets including many emerging ones (Table 1) will (1) improve discovery power for GWAS and PRS, (2) improve representation of African genomics in the global context, (3) provide a unique framework to examine a wide range of health indices in African populations, and (4) aid insights into the biological mechanisms and aetiology underlying disease risk in African populations, informing the wider application of potential preventative or therapeutic strategies.

### Barriers and potential clinical utility of PRS in African populations

Hundreds of PRS studies have been carried out including those on its clinical utility mainly in European populations [62–65]. A recent systematic review by Kumuthini and colleagues [65] shows a conflict claim for and against utility of PRS. This analysis did not discover published evidence of a PRS’s clear clinical usefulness, though they show numerous examples of near evidence of clinical utility and ample demonstration of clinical validity [65]. Conversely, there is also a growing number of investigations suggesting that PRS are not more predictive than standard of care [66], for example, two retrospective studies that integrated coronary disease PRS and found no and a modest statistically significant improvement in accuracy compared to use of the same models without the score [67, 68]. In the analysis of two US cohorts, Mosley and colleagues [68] show that the PRS was associated with incident of coronary heart disease events but did not significantly improve discrimination, calibration, or risk reclassification compared with conventional predictors. However, a few PRS-based genetic risk estimates from continental Africa [15, 16] have shown promises in the ability of PRS to identify subgroups of individuals who may benefit from the prioritisation of preventive actions.

The potential utility of PRS in African populations is limited by many factors. First, the current PRS methods limit the general utility of PRS as they have mostly been developed and optimised in European populations. Unless sufficient research is also undertaken to optimise the application of PRS in African populations, there is a risk of inequitable distribution of health benefits from future clinical utility of PRS. PRS calculations cannot, for now, capture the full spectrum of disease risk because of allele types, their frequencies, and their effect sizes. For precise estimates to be possible, a complete representation of all contributing loci is desirable [69]. Current PRS methods can be improved with non-genetic parameters included in the models. More dynamic methods to estimate the effects of specific genetic variations given the genetic, demographic, and clinical risk factor backgrounds of the individual are anticipated to be developed as representation from Africa and other underrepresented populations increases. It is reassuring to see a coordinated efforts such as the Polygenic RIsk MEthods in Diverse Populations (PRIMED) that promises to deliver new methods for risk prediction in diverse ancestry and specifically a pan-Africa initiative—CARdiometabolic Disorders IN African-ancestry PopuLations (CARDINAL) project which aim to test PRS performance on African individuals with phenotype and genotype data available from H3Africa projects [70].

Lack of infrastructure and difficulties with accurate phenotyping are major barriers for conducting genomic research in resource limit settings like Africa. However, to ensure collection of more accurate phenotype, a standardised data collection instrument known as the H3Africa Standard Case Report Form (CRF) was developed by H3Africa [71], which enables efficient and complete data collection, processing, analysis, and reporting. While the issue of heterogenous phenotyping remains, there exists some commonly statistical method for analysing a collection of studies for which the effect sizes are expected to vary. Random-effects model for GWAS meta-analysis is designed specifically for the case in which there is heterogeneity [72]. The other commonly used fixed-effects meta-analysis will only increase power if effects are homogeneous across studies.

For a sustainable solution to some lack of infrastructure in the continent for genomic research, H3Africa [60] and other initiatives in Africa are partnering with biotechnology company such as Illumina. Africa can now boast of large genomics facility with the latest cutting-edge technology in Nigeria, South Africa, Kenya, Uganda, and other places. Notably are African Center of Excellence for Genomics in Infectious Diseases (ACEGID) Nigeria, KwaZulu-Natal Research Innovation and Sequencing Platform (KRISP), Centre for Epidemic Response and Innovation (CERI), and Centre for Proteomic and Genomic Research (CPGR) in South Africa. These new facilities enable African researchers to avoid major delays in cross-border shipping of biological samples and to ensure the ability to reuse these valuable datasets. In the past, infrastructure for sample processing, biobanking, genotyping, or sequencing and computational analysis are often outsourced, but these are now gradually changing. To optimally benefit from these technologies that foster implementation of PRS, Africa countries must first address the collapsing primary health care mostly in the rural communities, although, in the urban context, Africans have choice to access advanced medical technologies despite the current shortcomings of the healthcare systems including an increased in demand for genetic tests for preventive purposes (for example: cancer panels such as MammaPrint in South Africa).

Even with the most accurate PRS, addition of conventional risk factors to PRS would be central to potential clinical utility in Africa [16, 73]. Such clinical utility of PRS in Africa will require an extensive awareness and education for both the physicians, patients, and the public regarding the importance and interpretation. In particular, methods that integrate uncertainty deriving from measured as well as unmeasured factors would be valuable for communicating the uncertainty associated with genetic risk estimations at an individual level [74, 75] including approaches to mitigate incidental findings, genetic discrimination, and what the role of counsellors and expert mediators would be in such cases. Such clinical utility of PRS would need to be fully supported by a robust ethical framework and effective regulatory system. For example, ethically, the genetic study of cognitive ability remains controversial both scientifically and ethically, and as such, the utility of PRS would need to be regulated within certain traits and phenotypes.

Ultimately, as shown by a few African PRS studies, PRS may have clinical utility in Africa when combined with traditional risk factors for some diseases, such as cardiometabolic traits, but first, right healthcare system and genomic infrastructure must be in place, and large-scale African genomic studies are required to demonstrate the utility of polygenic risk estimation. This might require the development of multiple models for every disease given the broad genetic diversity within Africa.

### Future directions and conclusion

PRS currently have limited transferability, caused mainly lack diversity in genomic studies. To improve the prediction accuracy of PRS in African ancestry individuals, it is most important to include ethnically diverse individuals from continental Africa in genomic studies. Wonkam [76] recommended a rough estimate of about three million African genomes (3MAG) to capture the full scope of Africa’s genetic variation and a representative human reference genome. This is mostly hindered by the lack of accurate population descriptions of African populations. Participants are mostly defined as per their geographic region or country, while it is well established that most countries are not homogeneous and can have profound genetic differences. Botswana, for example, hosts populations that are descendants of Bantu from West Africa and people of South African ancestry [77]. Similarly, Bantu speakers of Uganda contrast with non-Bantu speakers from the same country. Substantial genetic variations across regions of Africa must be carefully addressed for the integration of genomics data in health care. In a personal communication with Wonkam [76], he explained that 3MAG was a very rough estimate base on two simple assumptions: (1) the Human Genomes Project (HGP) estimated that between two unrelated individuals, there is a SNV every 1300 bp, therefore about 3,000,000 SNV difference, considering that each genome has 3 billion nucleotides. (2) owing to the great diversity in Africa, if we assume that most African has at least one uncaptured SNV, we need a minimum of 3 million African to capture, at least, the SNVs in our genome, although we see the potential of bias in this estimation [78] and several logistical and financial challenges to consider. Nevertheless, we agree with the proposition that a comprehensive and extensive genome sequencing programme in Africa is of utmost importance. This undertaking is essential for the comprehensive representation of the continent’s extensive genetic diversity. New initiatives in Africa, such as the ambitious plan to establish eight Genomics Centres of Excellence (GenCoE) across the continent, seek to revolutionise access to cutting-edge genomics technologies and reshape the continent’s response to some of its most pressing health challenges (https://www.nature.com/articles/d44148-023-00052-z). The initiative, which carries a significant price tag of US$200 million, is built from the 3MAG programme and seeks to obtain financial support from many sources globally. These sources include African governments, industrial partners, the US government, and other funding agencies. It is imperative that Africa actively participates in the genomic medicine revolution, ensuring that it does not lag behind in harnessing the transformative potential it offers.

Such large-scale African genomic studies like 3MAG can reveal novel genes including causal genetic variants not found in previous Eurocentric studies. In addition, it would offer the opportunity to develop regional PRS within Africa to cater for genetic differences within Africans which is even larger than between Africans and Eurasians. Invariably, this would largely solve many barriers poised by difference in allele frequencies, effect sizes, and LD patterns when developing PRS. Leveraging the greater genetic diversity in Africa, within representative genomic data from Africa, PRS derived from African population may be more predictive to all global populations [6].

Currently, PRS statistical models are trained with Eurocentric datasets. While representation of African genomics is being improved which might take decades, statistical model could be trained to estimate the projected effect sizes and allele frequency of those unknown African GWAS loci for genetic risk prediction. With current advances in machine learning and artificial intelligence, expanding the PRS models is a more practical solution to addressing the effect of genetics and its interaction with environmental exposure [79]. This method will require to be trained with different datasets. Such datasets for PRS across diverse ethnic groups in African populations have been highlighted in Table 1. Eventually, more dynamic methods for estimating effects associated with individual genetic variants given the individual’s genetic, demographic, and clinical risk factor background should be developed [79]. We think the future of PRS in diverse Africa population lies in the development of multiple PRS models per disease from African discovery datasets.

Considering the current poor state of many healthcare settings in Africa, even with best models and perfect PRS transferability, the prospect of clinical utility of PRS is slim in resource-limited medical settings. It is most likely that PRS would first be accessible across Africa via direct-to-consumer (DTC) company and specialist private hospitals for only those who could afford it, but there are concerns about ethical legal and social issues (ELSI) and how PRS will be regulated. Regulatory bodies should consider limiting power in the hands of PRS service providers to use their discretion to test and report any conditions or traits; otherwise, the easy access to PRS may also lead to inappropriate use and abuse. For example, the use of PRS for embryo selection, intelligence, and other psychiatric and socio-behavioural traits is strongly recommended to be restricted.

Collectively, in the future, with increased representation of Africans in genomics, sophisticated predictive PRS models which account for both genetic and non-genetic factors, it may well be possible for PRS to be utilised in the medical practice for some diseases with multiple polygenic scores generated for different diseases or traits in combination with conventional risk factors. This would need to be guided with robust ethical framework, but more translational research is needed.

### Supplementary Information

**Additional file 1: Table S1.** Different methods for the calculation of PRS and their relevant parameters.
