# Supplementary material for: Polygenic risk scores for disease risk prediction in Africa: current challenges and future directions
Source: Genome Med. 2023 Oct 30;15:87. doi: 10.1186/s13073-023-01245-9 (PMC10614359; doi:10.1186/s13073-023-01245-9)
Supplement: Supplementary file 1 — Additional file 1: Table S1. Different methods for the calculation of PRS and their relevant parameters. [file 13073_2023_1245_MOESM1_ESM.docx]

| **Table S1- Different methods for the calculation of PRS and their relevant parameters** | | | | | | |
| --- | --- | --- | --- | --- | --- | --- |
| **Methods** | **Model** | **Year** | **Accounting for LD** | **Comment** | **LD/Effect size/Allele frequency** | **Reference** |
| LdPred | Bayesian PRS | 2015 | Calculate LD from reference panel. | LD information from a reference panel requires that the reference panel be a good match for the population from which summary statistics were obtained; in the case of a mismatch, prediction accuracy might be compromised. | **Effect size** The estimated effect of each SNP in the study will depends on the extent of linkage disequilibrium (LD) with the causal sites. LD in Africa population are shorter than other populations.  **Linkage disequilibrium** The LD differences between discovery population and target population if not the same ancestry or similar may lead to variation in the estimated effect sizes and hence to variable phenotypic prediction accuracies   **Allele frequencies of causal variants** Allele frequencies of causal variants may be different for different LD particularly if the variant is common in the discovery dataset but rare in target dataset. Such variants are likely to have noisy effect size estimates which may after it inclusion in the best PRS. | <https://www.ncbi.nlm.nih.gov>/pmc/articles/PMC4596916/ |
| PRS-CS | Bayesian PRS | 2019 | Calculate LD from reference panel. | Continuous shrinkage priors enable conjugate block update of the SNP effect sizes in posterior inference and thus can accurately model local LD patterns and provide substantial computational improvements. |  | <https://www.nature.com/articles/s41467-019-09718-5> |
| PANPRS | Bayesian PRS | 2020 | Calculate LD from reference panel. | They present a unified framework to incorporate local LD pattern, multiple functional annotations and genetic pleiotropic information. |  | https://pubmed.ncbi.nlm.nih.gov/34483403/ |
| Multi-ethnic PRS | Bayesian PRS | 2018 | Calculate LD from reference panel and target data. | More accuracy is achieved with training data containing the same LD patterns as the target population. |  | https://www.ncbi.nlm.nih.gov/pmc/articles/PMC5726434/ |
| PRSice | Standard PRS | 2014 | LD Clumping | SNPs in linkage disequilibrium can be included or removed |  | <https://academic.oup.com/bioinformatics/article/31/9/1466/200539?login=true> |
| Lassosum | Bayesian PRS | 2017 | Calculate LD from reference panel. | PRS are constructed using summary statistics and a reference panel in a penalized regression framework. |  | https://onlinelibrary.wiley.com/doi/full/10.1002/gepi.22050 |
| POLARIS | Standard PRS | 2018 | Application of PCA | There is an adjustment for LD which includes a stabilization parameter to cope with cases of extreme LD. It adjusts for LD between SNPs and informs the analysis with previously reported effect sizes of a SNP's association with disease. |  | https://www.ncbi.nlm.nih.gov/pmc/articles/PMC6001515/ |
